# The Effectiveness of Early Enteral Nutrition on Clinical Outcomes in Critically Ill Sepsis Patients: A Systematic Review

**DOI:** 10.3390/nu15143201

**Published:** 2023-07-19

**Authors:** Sun Jae Moon, Ryoung-Eun Ko, Chi-Min Park, Gee Young Suh, Jinseub Hwang, Chi Ryang Chung

**Affiliations:** 1 Department of Medicine, Samsung Medical Center, Sungkyunkwan University School of Medicine, Seoul 06351, Republic of Korea; sunjae16.moon@samsung.com; 2 Department of Critical Care Medicine, Samsung Medical Center, Sungkyunkwan University School of Medicine, Seoul 06351, Republic of Korea; ryoungeun.ko@samsung.com (R.-E.K.);; 3 Department of Surgery, Samsung Medical Center, Sungkyunkwan University School of Medicine, Seoul 06351, Republic of Korea; 4 Division of Pulmonary and Critical Care Medicine, Department of Medicine, Samsung Medical Center, Sungkyunkwan University School of Medicine, Seoul 06351, Republic of Korea; 5 Department of Data Science, Daegu University, Gyeongsan 38453, Republic of Korea

**Keywords:** intensive care unit, enteral nutrition, sepsis, septic shock, systematic review

## Abstract

The optimal timing of enteral nutrition (EN) in sepsis patients is controversial among societal guidelines. We aimed to evaluate the evidence of early EN’s impact on critically ill sepsis patients’ clinical outcomes. We searched the MEDLINE, Embase, CINAHL, Cochrane Library, ClinicalTrials.gov, and ICTRP databases on 10 March 2023. We included studies published after 2004 that compared early EN versus delayed EN in sepsis patients. We included randomized controlled trials (RCTs), non-RCTs, cohort studies, and case–control studies. Forest plots were used to summarize risk ratios (RRs), including mortality and mean difference (MD) of continuous variables such as intensive care unit (ICU) length of stay and ventilator-free days. We identified 11 eligible studies with sample sizes ranging from 31 to 2410. The RR of short-term mortality from three RCTs was insignificant, and the MD of ICU length of stay from two RCTs was −2.91 and −1.00 days (95% confidence interval [CI], −5.53 to −0.29 and −1.68 to −0.32). Although the RR of intestinal-related complications from one RCT was 3.82 (95% CI, 1.43 to 10.19), indicating a significantly higher risk for the early EN group than the control group, intestinal-related complications of EN reported in five studies were inconclusive. This systematic review did not find significant benefits of early EN on mortality in sepsis patients. Evidence, however, is weak due to inconsistent definitions, heterogeneity, risk of bias, and poor methodology in the existing studies.

## 1. Introduction

Sepsis is a life-threatening condition that occurs when the host immune system fails to control an infection and triggers a systemic inflammatory response, leading to organ dysfunction and multiple organ failure. [1]. Sepsis induces the production of inflammatory cytokines that mediate the degradation of muscle proteins, the resorption of bone tissue, and the lipolysis of adipocytes [2]. This leads to accelerated and pronounced muscle atrophy, predominantly in the early phase of sepsis [3,4]. Consequently, sepsis survivors often suffer from intensive care unit (ICU)-acquired weakness or functional impairment associated with this pathophysiology and ICU-related immobilization [5].

Sepsis patients often require vasopressor support that diverts blood flow from the splanchnic circulation to other vital organs, potentially compromising intestinal perfusion. Therefore, initiating early enteral nutrition (EN) may pose a risk of intestinal-related complications, such as nonocclusive mesenteric ischemia and bowel necrosis [6,7]. Medical guidelines offer no consensus regarding the optimal timing of EN in critically ill sepsis patients. The Surviving Sepsis Campaign (SSC)-3 has suggested starting EN within 24 h of sepsis or septic shock [8]. In contrast, the American Society for Parenteral and Enteral Nutrition/Society of Critical Care Medicine (ASPEN/SCCM) advised delaying EN until the patient achieves hemodynamic stability [9]. The European Society of Parenteral and Enteral Nutrition (ESPEN) did not specify an explicit criterion for the timing of EN in sepsis patients [10]. However, the ESPEN recommended that early EN should be initiated in septic patients after hemodynamic stabilization. In contrast, the Japanese Clinical Practice Guideline for Management of Sepsis and Septic Shock (J-SSCG 2020) recommended initiating EN in the acute phase of sepsis (within 24 to 48 h after critical illness treatment) [11]. Lastly, the recent SSC-4 guidelines advocated for early EN within 72 h of ICU admission [12]. Such different guidelines on the timing of EN in sepsis patients have highlighted the controversy between early and delayed EN approaches.

This study aims to perform a comprehensive and updated systematic review of the available evidence, including the most recent research findings, on the effect of early EN on clinical outcomes, such as mortality and intestinal-related complications, in critically ill sepsis patients.

## 2. Materials and Methods

### 2.1. Study Design

We conducted this systematic review according to the Preferred Reporting Items for Systematic Reviews and Meta-Analyses guidelines [13]. The research protocol was submitted and registered before the start of the study in the Prospective Register of Systematic Reviews (PROSPERO) (CRD42022299216).

### 2.2. Study Selection and Databases

The MEDLINE, Embase, Cumulative Index to Nursing and Allied Health Literature, and Cochrane Central Register of Controlled Trials were selected as the core databases. ClinicalTrials.gov and the International Clinical Trials Registry Platform were chosen as additional databases, and the references recommended by the five guidelines related to early EN were also listed for screening [8,9,10,11,12]. The publication date was limited to the year the SSC guideline was first published or thereafter; studies published between 1 January 2004 and 10 March 2023 were searched using the filter. Titles and abstracts were reviewed to identify those that qualified for full-text evaluation after eliminating duplicates. The keywords used were “enteral feeding”, “sepsis”, and “intensive care”. Following a review of the final search queries by the librarian, a search was conducted by one author (S.J.M.) on 10 March 2023. All search queries are presented in Table S1. Inclusion criteria were as follows: (1) the study was conducted in critically ill adults aged ≥18 years, and the number of patients with sepsis comprised ≥50% of the total included patients in the intensive care unit (ICU) setting [8,12,14,15]; (2) the timepoint for early EN administration was specified; (3) the study compared outcomes in the delayed EN group following early EN in the control group; (4) the study was published after 2004 (when the first edition of SSC was published) [14]; (5) the study was a randomized controlled trial (RCT), nonrandomized controlled trial, cohort, or case–control design. The exclusion criteria were as follows: (1) pediatric critical care study; (2) a study in which only parenteral nutrients (PN) were administered without EN (as a control group); (3) studies with only abstracts or proceedings (after contacting the authors to confirm that they did not publish the original research). Data were extracted after two authors (S.J.M. and R.-E.K.) independently screened the titles and abstracts according to predetermined inclusion criteria. If there were discrepancies, the process was repeated until a consensus was reached. A third author (C.R.C.) was consulted if there were discrepancies.

### 2.3. Data Extraction

Before beginning the study, the items to be extracted were selected and extracted using a unified form. Information on sex, age, and body mass index (BMI) was extracted as baseline characteristics of the studies. Information on the country in which the study occurred was extracted. Regarding sepsis-related extraction items, information on the initial severity score (i.e., Sequential Organ Failure Assessment [SOFA] mean score) was extracted along with information on the etiology of sepsis, vasopressor use, and mechanical ventilator support. The sample size of each study’s intervention, cohort, case, and control groups was extracted, and the intention-to-treat concept for RCTs was extracted from the sample size information. Data on the timing of EN start, the actual delivered volume or energy of EN for each group, and information on whether or not PN was supplied during EN were extracted [16,17]. Outcome information included mortality (no time limit), SOFA score change, length of stay, and duration of organ dysfunction (i.e., duration of mechanical ventilation) [18]. Information on EN-related adverse events, intestinal complications, and nosocomial infection was also collected [19]. Two authors (S.J.M. and R.-E.K.) performed all extraction processes independently. Any discrepancies were resolved among the authors, as previously described.

The information was categorized on the basis of type. The input variables for the categorical variables were organized in a two-by-two table. For continuous variables, means and standard deviations were calculated as suggested by the Cochrane Handbook for Systematic Reviews [20].

### 2.4. Risk of Bias

Quality assessment was conducted using the Cochrane Risk-of-Bias 2 (Cochrane RoB-2) tool for RCTs [21] and the Newcastle–Ottawa scale for cohort or control studies [22]. We used the Cochrane RoB-2 tool to systematically assess the risk of bias in RCTs, which had domains for trial design, conduct, and reporting [21]. We also used the Newcastle–Ottawa scale to evaluate the quality of cohort and case–control studies, which had common domains for selection and comparability, and specific domains for outcome (cohort studies) and exposure (case–control studies) [22]. The selection domain examined the suitability and representativeness of the cohort or case definitions and the reasonableness of the control group’s definition and selection criteria [22]. The comparability domain checked whether matching was used and whether confounders were well-adjusted [22]. The outcome domain, applicable only to cohort studies, assessed the appropriateness of outcome variables and the follow-up period [22]. The exposure domain, applicable only to case–control studies, assessed the ascertainment of exposure and nonresponses [22]. The authors (S.J.M. and R.-E.K.) independently performed quality assessments. Other authors (C.-M.P. or C.R.C.) were consulted to identify any discrepancies.

### 2.5. Synthesis of Results

The risk ratio (RR) for mortality and adverse events (i.e., vomiting or intestinal ischemia) and mean difference for continuous variables, such as ICU length of stay (LOS) and ventilator-free days were used to display forest plots [20]. Due to significant heterogeneity, indicators that were difficult to integrate were narrated and presented in tables. All effective sizes were reported using 95% confidence intervals (CIs). All analyses were performed using the Review Manager 5.4 (Cochrane Collaboration, Oxford, UK). The CI was presented as forest plots for a pooled effect size of 95%.

## 3. Results

### 3.1. Flow of Studies

**Figure 1:**
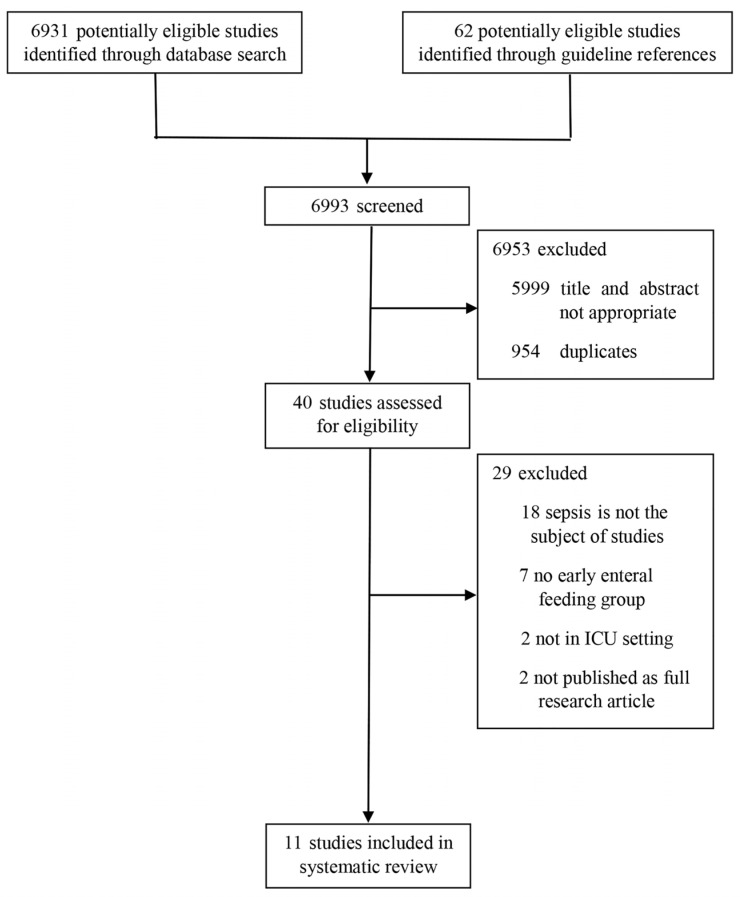
Flowchart of the systematic review process.

In total, 6931 studies were retrieved from three core databases, and 62 were recruited from additional databases (ClinicalTrials.gov and the International Clinical Trial Registry Platform). A total of 6993 publications were screened. We excluded 954 duplicates, and an additional 5999 were excluded after independent screening by two authors according to the inclusion and exclusion criteria, resulting in 40 studies being assessed for eligibility. Of those, 29 were excluded; 18 were excluded because they did not investigate sepsis, seven had no early EN group, two did not regard critically ill patients, and two were not published as full research articles (Figure 1) [23,24,25,26,27,28,29,30,31,32,33]. Finally, 11 studies were included in the systematic review.

### 3.2. Clinical Characteristics of Included Studies

Among the 11 studies that met the inclusion criteria, three were RCTs, three were cohort studies, and the remaining five were case–control studies; six were performed in Asian countries, along with four in the US, and one in Europe. The studies’ sample size varied widely, ranging between 31 and 2410.

The mean age of the patients also varied, ranging between 44.33 and 71.66 years, while the mean BMI ranged between 20.20 and 37.60. Considering the etiology of sepsis, four of the 11 studies were on pneumonia; furthermore, three were on abdominal infection, along with one on trauma, one on pulmonary and abdominal infection, one on soft-tissue infection, and one with no specific etiology. Among all the included studies, the mean SOFA score ranged between 7.70 and 11.00, and five studies included patients who supported both vasopressor and mechanical ventilation (Table 1).

### 3.3. Nutrition Characteristics of Included Studies

As for the start timing of early EN, seven of 11 studies started immediately (0 h). Among RCTs, Patel et al. [29] and Sun et al. [32] defined early EN as EN that started within 24–48 h of ICU admission, and Reignier et al. [30] defined early EN as EN initiated within 24–96 h of ICU admission. (Table 2). Five of the 11 studies reported the average daily amount of energy supplied (Table 2). The information on PN was reported in 10 of 11 studies. Three studies reported that all patients supported PN, and one study presented that PN was supported only in the delayed EN group. Six studies reported that they did not support PN in all studied patients (Table 2).

### 3.4. Clinical Outcomes of Included Patients

**Figure 2:**
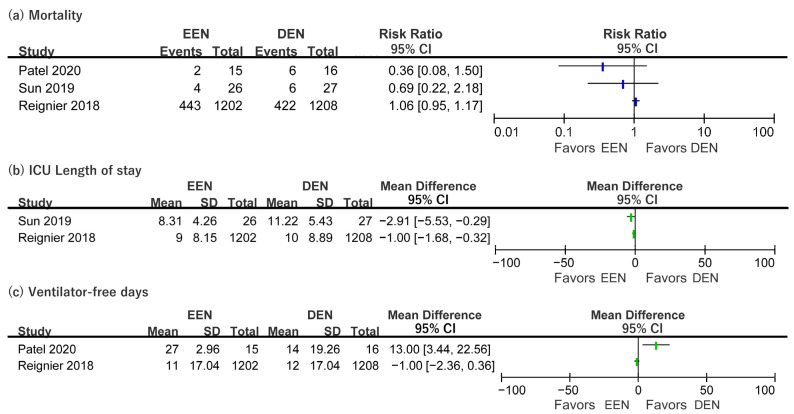
Forest plot of clinical outcomes (**a**) mortality; (**b**) length of stay in intensive care unit; (**c**) ventilator-free days. CI, confidence interval; DEN, delayed enteral nutrition; EEN, early enteral nutrition; ICU, intensive care unit; SD standard deviation. The blue bars in the figure indicate the results of the risk ratio and the green bars indicate the results of the mean difference [29,30,32].

For short-term mortality, the RR in Patel et al. [29], Sun et al. [32], and Reignier et al. [30] ranged from 0.36 to 1.06 (Figure 2). The 90 day mortality was only reported in Reignier et al. [30], but the difference between the early and delayed EN groups was not significant (Table 3). Two cohort studies that evaluated 60 day mortality had conflicting results. Ortiz Reyes et al. [27] reported that 60 day mortality was not significantly different between early and delayed EN groups (Table 3). However, the early EN group had significantly reduced 60 day mortality (37% vs. 53%, *p* = 0.039) in Jiang et al. [24] (Table 3). ICU mortality was reported by Reignier et al. [30], Ortiz Reyes et al. [27], and Koga et al. [25]. ICU mortality ranged from 12% to 33% for early EN and from 20% to 33% for delayed EN, but the difference between groups was insignificant (Table 3). The mean difference in the ICU LOS from Sun et al. [32] and Reignier et al. [30] was −2.91 (95% CI −5.53–−0.29) and −1.00 (−1.68–−0.32), respectively (Figure 2). The mean difference in SOFA score changes (48–72 h) in Patel et al. [29] and Sun et al. [32] was −5.00 (95% CI = 14.91–−4.91) and −0.99 (−2.37–−0.39), respectively. Patel et al. [29] and Reignier et al. [30] presented ventilator-free days. Patel et al. [29] showed that early EN significantly increased the mean difference of ventilator-free days between early EN and delayed EN groups. However, Reignier et al. [30] did not show a significant mean difference in ventilator-free days between the two groups. The RR for ventilator-associated pneumonia was derived from Patel et al. [29] and Reignier et al. [30], but the RRs were not significant in either study (Figure S1). The details of the clinical outcomes are also presented in Table S2.

Intestinal-related complications of EN were reported in five studies (two RCTs, two cohorts, and one case–control study). Patel et al. [29] and Reignier et al. [30] reported vomiting and intestinal ischemic events as adverse events. The RR of intestinal ischemia was 3.82 (95% CI, 1.43–10.19), which was derived from Reignier et al. [30]. However, Patel et al. [29] did not show a difference in intestinal ischemic events between the two groups. The risk of vomiting was higher in the early EN group than in the delayed EN group (RR = 2.12, 95% CI 1.78–2.51) in Reignier et al. [30]. However, Patel et al. [29] showed no significant difference in risk of events occurring between the two groups (Figure S1). The details of the GI complications and tolerance are presented in Table 4.

### 3.5. Risk of Bias

Considering the quality assessment, all three RCTs had a risk-of-bias concern in the performance bias items, which assess whether the personnel involved in providing care, other than the intervention, were aware of the allocated interventions. Due to the nature of the early EN intervention, it was impossible to blind the providers, which could have influenced their behavior or decisions. Detailed information on the quality assessment for each study design, including the risk-of-bias domains and the scoring criteria, can be found in Tables S3–S5.

## 4. Discussion

The results of the three RCTs [29,30,32] showed that early EN did not have significant effects on either mortality or clinical outcomes, except for intestinal-related complications. Although the RR of intestinal-related complications from one RCT indicated a significantly higher risk for the early EN group than for the control group, intestinal-related complications of EN reported in five studies were inconclusive.

These findings align with the recommendations of the 2016 ASPEN/SCCM and 2019 ESPEN guidelines, which advised caution in initiating early EN for sepsis patients. The 2016 ASPEN/SCCM guideline recommended early EN for sepsis patients with caution, but did not include any RCTs on this topic [9]. The 2019 ESPEN guideline (Recommendation 44) provided a more comprehensive review of early EN in sepsis patients and included two RCTs in this recommendation [30,34]. The guidance reported that early EN initiated within 36 h did not significantly reduce mortality or infection rates, but did significantly increase intestinal related complications [10]. However, systematic literature reviews that analyzed all critically ill patients, not only those with sepsis, found more benefit than harm from early EN [11,12]. These discrepancies among recommendations may have been influenced by the study populations in the included studies and different definitions and outcomes related to early EN.

Septic shock is a severe form of sepsis characterized by hypotension, vasopressor support, and tissue hypoperfusion [1]. In the early phase of septic shock, blood flow is diverted from nonessential splanchnic circulation to vital organs, such as the brain, lungs, and heart. This may result in intestinal dysfunction, which is thought to play a key role in developing sepsis-related multiorgan failure by increasing intestinal permeability, bacterial translocation, and systemic inflammation [35]. However, the exact mechanisms and outcomes of septic shock-induced intestinal dysfunction, such as impaired intestinal perfusion, increased intestinal permeability, bacterial translocation, and bowel ischemia and necrosis, are still poorly understood. The NUTRIREA-2 trial is a well-designed, pragmatic, large-scale, multicenter clinical trial implemented to compare the effects of early EN versus early parenteral nutrition on mortality and morbidity in patients with shock. A recent post hoc analysis of the NUTRIREA-2 trial reported that the incidence of intestinal ischemia was 1% in critically ill ventilated patients with shock [7]. Moreover, EN, dobutamine use, higher severity score, and lower hemoglobin level were independent risk factors for acute intestinal ischemia [7]. These results suggest that early EN may increase the risk of acute intestinal ischemia in septic shock patients. However, to draw definitive conclusions, more detailed clinical data are required along with standardized timing and outcome definitions of early EN and intestinal ischemia in sepsis patients. Clinical heterogeneity was often observed among the studies on this topic of early EN. These studies differed in the timing of EN initiation, the use of PN support, and the amount of total calorie provision. For a clear conclusion on the effects of early EN in sepsis patients, standardized protocols are needed for RCTs on this topic.

We identified an inaccurate description of the NUTRIREA-2 trial in the SSC-4. The SSC-4 stated that early EN timing from the NUTRIREA-2 trial was within 72 h of ICU admission [12]. However, this study randomized patients after 24 h of ICU admission and started EN within 72 h after randomization. Therefore, the correct timing of EN initiation in the NUTRIREA-2 trial was within 96 h of ICU admission (Table 2).

The 2019 ESPEN guideline recommends initiating early EN after achieving hemodynamic stability [10]. However, the RCTs included in this analysis defined early EN according to the time of ICU admission, not on hemodynamic stability, as suggested by the SSC-4 guideline. This may be due to the difficulty of clearly defining hemodynamic stability in critically ill patients. Some studies have used the vasoactive–inotropic score to define hemodynamic stability for early EN, but a general consensus has yet to be formed [36,37]. Therefore, further studies are warranted to establish a definition of hemodynamics and to evaluate the efficacy of the early criteria based on the 2019 ESPEN guideline.

While some guidelines recommend early EN in sepsis according to the analysis of all critically ill patients, we performed a systematic review of the effects of early EN in sepsis patients only, including recent RCTs. To mitigate the limited number of RCTs on this topic, we also included studies of other designs, such as cohort and case–control. In addition, we synthesized the results of multiple indicators in different settings, including the effects on the timing of EN initiation and clinical outcomes. There is an emerging consensus on the outcome measures of nutritional intervention studies [38]. Researchers in the field of early EN in critically ill patients should strive to report standardized outcomes and safety profiles with consistent definitions and references [19].

This study had some considerable limitations. Firstly, the definition of early EN was not uniform across the studies in our systematic review, despite applying a rigorous definition of time zero as being at ICU admission. This may be due to the lack of agreement on what constitutes “early”, whether it should be based on time or on hemodynamic stability, and, if based on time, what the cutoff point should be. Secondly, blinding was difficult due to the nature of the interventions. Therefore, most studies were nonrandomized and prone to both measured and unmeasured confounding factors. Moreover, the included studies had different definitions and measurements of early EN as the primary outcome. These aspects should also be standardized for generating high-quality evidence in the future. Lastly, we did not perform a meta-analysis due to the high heterogeneity among the three RCTs and the need for a consistent time threshold for initiating EN. Unfortunately, no firm conclusions could be drawn due to inconsistent definitions, heterogeneity, risk of bias, and poor methodology in the initially proposed meta-analysis studies. Accordingly, this systematic review study finally included only three trials assessing mortality and intestinal-related complications.

## 5. Conclusions

This systematic review did not find significant or clear benefits of early EN on mortality in sepsis patients, compared to delayed EN. Future guidelines should address the heterogeneity in intervention protocols and establish the criteria for early EN on the basis of the results of multicenter RCTs in homogeneous populations.

## Figures and Tables

**Table 1:** Baseline characteristics of 11 studies.

	Study Design	Country	Sample Size	Mean Age (Years)	Mean BMI (m^2^/kg)	Main Sepsis Etiology	Mean SOFA Score	Vasopressor/MV Support
Ortiz-Reyes et al., 2022 [27]	Prospective cohort	USA	626	57.70	28.20	Pneumonia	9.40	+/+ *
Patel et al., 2020 [29]	RCT	USA	31	59.87	32.71	Pneumonia	10.52	+/not reported
Liu et al., 2020 [26]	Case–control	China	63	47.84	Not reported	Pneumonia	8.97	+/+
Jiang et al., 2020 [24]	Prospective cohort	China	163	70.09	20.20	Abdominal infection	9.85	+/+
Sun et al., 2019 [32]	RCT	China	53	58.06	24.74	Abdominal infection	9.26	All not reported
Reignier et al., 2018 [30]	RCT	France	2410	66.00	27.85	Not reported	11.00	+/+
Koga et al., 2018 [25]	Retro-prospective cohort	Japan	173	44.39	21.59	Pneumonia (EEN) and abdominal infection (DEN)	8.96	+/not reported
Haac et al., 2018 [23]	Case–control	USA	85	59.72	37.60	Necrotizing soft-tissue infection	7.70	+/not reported
Sun et al., 2017 [31]	Case–control	China	82	71.66	22.91	Abdominal infection	8.00	All not reported
Patel et al., 2016 [28]	Case–control	USA	52	58.29	28.00 (trophic EEN), 26.55 (full EEN)	Pneumonia	Not reported	+/+
Yuan et al., 2011 [33]	Case–control	China	82	44.33	20.70	Trauma	Not reported	Not reported/+

* Indicates whether vasopressor/MV support has been applied (+) at the baseline. BMI, body mass index; DEN, delayed enteral nutrition; EN, enteral nutrition; EEN, early enteral nutrition; MV, mechanical ventilation; RCT, randomized controlled trial; SOFA, Sequential Organ Failure Assessment.

**Table 2:** Nutrition characteristics of 11 included studies.

	Timing of EEN/DEN Delivery (Range Hours after ICU Admission)	Actual Delivered Energy of EEN/DEN Group (Mean kcal/Day or kcal/kg/Day)	PN Support of EEN/DEN Group (+ or −)
RCT			
Patel et al., 2020 [29]	24 to 48 h/after 48 h	252/307 kcal/day (7 days)	−/− *
Sun et al., 2019 [32]	24 to 48 h/after 96 h	Not reported	−/−
Reignier et al., 2018 [30]	24 to 96 h/after 96 h	1413/1552 kcal/day (7 days)	−/+
Cohort			
Ortiz-Reyes et al., 2022 [27]	0 to 48 h/after 48 h	993/772 kcal/day (12 days)	+/+
Jiang et al., 2020 [24]	0 to 24 h/after 24 h	Not reported	Not reported
Koga et al., 2018 [25]	0 to 48 h/after 48 h	10.4/1.4 kcal/kg/day (7 days)	+/+
Case–control			
Liu et al., 2020 [26]	0 to 48 h/after 48 h	Not reported	−/−
Haac et al., 2018 [23]	0 to 48 h/after 48 h	Not reported	−/−
Sun et al., 2017 [31]	48 to 72 h/after 96 h	Not reported	−/−
Patel et al., 2016 [28]	0 to 48 h/after 48 h	329 and 778/307 kcal/day (7 days) (trophic EEN and full EEN/DEN)	−/−
Yuan et al., 2011 [33]	0 to 336 h/after 336 h	Not reported	+/+

* Indicates whether parenteral nutrition was provided (+) or not (−) during administrating enteral nutrition in EEN/DEN groups. DEN, delayed enteral nutrition; EEN, early enteral nutrition; ICU, intensive care unit; PN, parenteral nutrition; RCT, randomized controlled trial.

**Table 3:** Long-term (more than 30 days) and ICU mortality outcomes from one RCT and three cohort studies.

	Definition of Mortality	EEN Group % (Events/Total)	DEN Group % (Events/Total)	Reported Significance, *p*
RCT				
Reignier et al., 2018 [30]	Day 90 mortality	45% (530/1185)	43% (507/1192)	0.28
	ICU mortality	33% (429/1202)	31% (405/1208)	0.17
Cohort				
Ortiz-Reyes et al., 2022 [27]	Day 60 mortality	40% (211/526)	45% (45/100)	0.36
	ICU mortality	31% (161/526)	33% (33/100)	0.55
Jiang et al., 2020 [24]	Day 60 mortality	37% (31/85)	53% (41/78)	0.039 *
Koga et al., 2018 [25]	ICU mortality	12% (9/78)	20% (23/113)	0.11

* Significant difference between EEN and DEN groups. DEN, delayed enteral nutrition; EEN, early enteral nutrition; ICU, intensive care unit; RCT, randomized controlled trial.

**Table 4:** Detailed information on GI complications and tolerance of enteral feeding (five studies reported, sorted by the study design).

	GI Complications (EEN/DEN Group)	Tolerance of EN (EEN/DEN Group)
RCT		
Patel et al., 2020 [29]	Vomiting within 72 h: 13% (EEN), 50% (DEN) Vomiting within seven days: 20% (EEN), 56% (DEN) * Ileus within seven days: 0% (EEN), 0% (DEN) Intestinal ischemia within 30 days: 0% (EEN), 0% (DEN) Small bowel obstruction within 30 days: 0% (EEN), 0% (DEN)	Episode of GRV more than 500 mL: 0% (EEN), 0% (DEN)
Reignier et al., 2018 [30]	Vomiting within 28 days: 34% (EEN), 24% (DEN) * Diarrhea within 28 days: 36% (EEN), 33% (DEN) * Intestinal ischemia within 28 days: 2% (EEN), less than 1% (DEN) * Acute colonic pseudo-obstruction within 28 days: 1% (EEN), 1% (DEN) *	Not reported (GRV were not monitored)
Cohort		
Ortiz-Reyes et al., 2022 [27]	Vomiting within 28 days: 10.5% (EEN), 13.0% (DEN) Diarrhea within 28 days: 2.3% (EEN), 3.0% (DEN) Subjective discomfort within 28 days: 0.8% (EEN), 0% (DEN) Intestinal ischemia (necrotic) within 28 days: 0.3% (EEN), 0% (DEN) (significance not reported)	High GRV: 7.2% (EEN), 7.4% (DEN)
Jiang et al., 2020 [24]	Mean global AGI grade: 1.3 (EEN), 1.6 (DEN)	Mean GRV: 98.6 mL (EEN), 77.7 mL (DEN)
Case-control		
Patel et al., 2016 [28]	Ileus: 2.7% (trophic EEN), 7.1% (full-calorie EEN), and 6.7% (DEN) (significance not reported) Nonocclusive intestinal ischemia or necrosis: 0% (EEN), 0% (DEN)	Feeding tolerance: 97.3% (trophic EEN), 85.7% (full-calorie EEN), and 86.6% (DEN)

* Significant difference between EEN and DEN groups. AGI, acute gastric injury; DEN, delayed enteral nutrition; EEN, early enteral nutrition; EN, enteral nutrition; GI, gastrointestinal; GRV, gastric residual volume; RCT, randomized controlled trial.

## Data Availability

The dataset used and analyzed during the current study is available from the corresponding author on reasonable request.

## Supplementary Materials

The following supporting information can be downloaded at https://www.mdpi.com/article/10.3390/nu15143201/s1: Table S1. Search queries; Table S2. Detailed information on mortality outcomes from each RCT and cohort study; Table S3. Summary of quality assessment of RCTs (three studies); Table S4. Quality assessment of cohort studies (three studies); Table S5. Quality assessment of case–control studies (five studies); Figure S1. Forest plot of vomiting and ventilator-associated pneumonia events from two RCTs.

## Abbreviations

BMI: body mass index; EN: enteral nutrition; ICU: intensive care unit; LOS: length of stay; RCT: randomized controlled trial; SOFA: Sequential Organ Failure Assessment; SSC: Surviving Sepsis Campaign.
