# Supplementary material for: The Effectiveness of Early Enteral Nutrition on Clinical Outcomes in Critically Ill Sepsis Patients: A Systematic Review
Source: Nutrients. 2023 Jul 19;15(14):3201. doi: 10.3390/nu15143201 (PMC10383540; doi:10.3390/nu15143201)
Supplement: Supplementary file 1 [file nutrients-15-03201-s001.zip › nutrients-2521325-supplementary.pdf]

Table S1. Search queries.

Database	Embase & MEDLINE Complete (2023.03.10 07:29 GMT)	
#1	'septic shock'/mj OR 'systemic inflammatory response syndrome'/mj OR 'multiple organ failure'/mj OR 'bacteremia'/mj OR 'endotoxic shock'/mj OR systemic-inflammatory-response* OR multi-system-inflammatory* OR systemic-inflammation-syndrome* OR hyperinflammatory-syndrom* OR sepsis-associated-hypotension* OR septicaemic-shock* OR septicemic-shock* OR endotoxin-shock* OR toxic-shock* OR multiple-organ-fail* OR multiple-organ-dysfunct* OR multiorgan-fail* OR multiorgan-dysfunct* OR multisystem-organ-fail* OR multisystem-dysfunct* OR bacteremi* OR fungemi* OR septic* OR sepsi* OR urosepsi* OR shock*	804,707
#2	'enteric feeding'/mj OR enteric-feed* OR enteral-nutrit* OR intragastric* OR nasogastric* OR nasojejunal* OR intestinal-feed* OR tube-feed* OR orogastric* OR orojejunal*	113,827
#3	'intensive care unit'/mj OR 'extracorporeal oxygenation'/mj OR 'vasoconstrictor agent'/mj OR 'cardiotonic agent'/mj OR 'renal replacement therapy'/mj OR 'ventilator'/mj OR intensive-care* OR critical-care* OR ICU OR CCM OR stroke-unit* OR coronary-care-unit* OR burn-unit* OR close-attention-unit* OR intensivist* OR critical-ill* OR critically-ill*	1,088,176
#4	#1 AND #2 AND #3	3,737
#5	#4 AND [2004-2023]/py	3,157
**Database**	**CINAHL Complete (2023.03.10 08:30 GMT)**	
#1	(MM "Shock, Septic") OR (MM "Systemic Inflammatory Response Syndrome") OR (MM "Multiple Organ Dysfunction Syndrome") OR (MM "Bacteremia") OR systemic-inflammatory-response* OR multi-system-inflammatory* OR systemic-inflammation-syndrome* OR hyperinflammatory-syndrom* OR sepsis-associated-hypotension* OR septicaemic-shock* OR septicemic-shock* OR endotoxin-shock* OR toxic-shock* OR multiple-organ-fail* OR multiple-organ-dysfunct* OR multiorgan-fail* OR multiorgan-dysfunct* OR multisystem-organ-fail* OR multisystem-dysfunct* OR bacteremi* OR fungemi* OR septic* OR sepsi* OR urosepsi* OR shock*	184,638
#2	(MM "Enteral Nutrition") OR (MM "Enteral Feeding Pumps") OR (MM "Feeding Tubes") OR enteric-feed* OR enteral-nutrit* OR intragastric* OR nasogastric* OR nasojejunal* OR intestinal-feed* OR tube-feed* OR orogastric* OR orojejunal*	33,488
#3	(MM "Intensive Care Units") OR (MM "Critical Care") OR (MM "Critical Care Nursing") OR (MM "Extracorporeal Membrane Oxygenation") OR (MM "Vasoconstrictor Agents") OR (MM "Cardiotonic Agents") OR (MM "Renal Replacement Therapy") OR (MM "Continuous Renal Replacement Therapy") OR (MM "Ventilators, Mechanical") OR intensive-care* OR critical-care* OR ICU OR CCM OR stroke-unit* OR coronary-care-unit* OR burn-unit* OR close-attention-unit* OR intensivist* OR critical-ill* OR critically-ill*	429,965
#4	#1 AND #2 AND #3	3,247
#5	#4 AND 20040101-20231231	2,814
**Database**	**ClinicalTrials.gov (2023.03.10 07:42 GMT)**	
#1	septic OR sepsis OR urosepsis OR uroseptic OR shock OR shocks OR bacteremia OR bacteremic OR fungemia OR fungemic OR "SIRS" OR	4,551

	"MODS"	
#2	(enteric AND feed) OR (enteral AND feed) OR (enteral AND nutrit) OR intragastric OR nasogastric OR nasojejunal OR tube-feed OR orogastric OR orojejunal	2,212
#3	#1 AND #2	96
#4	#3 AND 20040101-20231231	95
**Database**	**International Clinical Trials Registry Platform (ICTRP) (2023.03.10 07:53 GMT)**	
	(septic OR sepsis OR urosepsis OR uroseptic OR shock OR shocks OR bacteremia OR bacteremic OR fungemia OR fungemic OR "SIRS" OR "MODS") AND ((enteric AND feed) OR (enteral AND feed) OR (enteral AND nutrit) OR intragastric OR nasogastric OR nasojejunal OR tube-feed OR orogastric OR orojejunal)	24
**Database**	**Cochrane Central Register of Controlled Trials (CENTRAL) (2023.03.10 07:56 GMT)**	
	(septic OR sepsis OR urosepsis OR uroseptic OR shock OR shocks OR bacteremia OR bacteremic OR fungemia OR fungemic OR "SIRS" OR "MODS") AND ((enteric AND feed) OR (enteral AND feed) OR (enteral AND nutrit) OR intragastric OR nasogastric OR nasojejunal OR tube-feed OR orogastric OR orojejunal)	840

CINAHL, Cumulative Index to Nursing and Allied Health Literature; GMT, Greenwich Mean Time.

Table S2. Detailed information on day-related outcomes from each RCT and cohort studies (three studies each).

	Timing of early EN	Days related outcome variables	EEN	DEN	Significance
**RCT**					
Patel 2020	24 to 48 h	ICU free days (30 days)	25 (14-27)	12 (0-22)	p = 0.014
		VFD (30 days)	27 (24-28)	14 (0-26)	p = 0.0090
Sun 2019	24 to 48 h	ICU days	8 (4)	11 (5)	p = 0.035
		Mechanical ventilation days	5 (3)	7 (4)	p = 0.0060
Reignier 2018	24 to 96 h	ICU LOS	9 (5-16)	10 (5-17)	NS
		Acute-care hospital LOS	17 (8-32)	18 (9-33)	NS
		Days without mechanical ventilation	11 (0-23)	12(0-23)	NS
		Days without vasopressor support (28 days)	20 (0-25)	21 (0-26)	NS
		Days without dialysis (28 days)	27 (0-28)	27 (0-23)	NS
**Cohort**					
Ortiz-Reyes 2022	0 to 48 h	ICU LOS in survivors	14 (11)	20 (14)	p = 0.00010
		Hospital LOS amongst survivors	23 (13)	28 (15)	p = 0.020
		ICU free days (28 days)	9 (9)	6 (8)	p = 0.00020
		VFD (28 days)	12 (10)	10 (9)	NS
		Length of MV in survivors	10 (11)	14 (15)	p = 0.00020
		Days alive and vasopressor free days (28 days)	17 (10)	16 (9)	p = 0.050
		Days alive and free of vasopressor (12 days)	7 (3)	6 (3)	p = 0.0070
		Time from ICU admission until discontinuation of vasopressor (28 days)	6 (5)	8 (6)	p = 0.00080
		PODs-free days (28 days)	11 (10)	9 (9)	p = 0.030
Jiang 2020	0 to 24 h	ICU LOS	11 (8-22)	10 (6-16)	p = 0.022
		Hospital LOS	23 (14-53)	18 (10-39)	p = 0.023
Koga 2018	0 to 48 h	Not reported	N/A	N/A	N/A

DEN, Delayed Enteral Nutrition; EEN, Early Enteral Nutrition; ICU, Intensive Care Unit; LOS, Length of Stay; N/A, Not Applicable; NS, Not Significant; PN, Parenteral Nutrition; PODs, Persistent Organ Dysfunctions; RCT, Randomized Controlled Trials; VFD, Ventilator Free Day(s).

Table S3. Summary of quality assessment of RCTs (three Studies)

	Domain 1. Risk of bias arising from the randomization process	Domain 2-1: Risk of bias due to deviations from the intended interventions	Domain 2-2: Risk of bias due to deviations from the intended interventions	Domain 3: Missing outcome data	Domain 4: Risk of bias in measurement of the outcome	Domain 5: Risk of bias in selection of the reported result
Patel 2020	Low	Unclear	High	Low	Low	Low
Sun 2019	Low	Unclear	High	Low	Low	Unclear
Reignier 2018	Low	Low	High	Low	Low	Low

RCTs, randomized controlled trials.

Table S4. Quality assessment of cohort studies (three studies).

	**Selection**				**Comparability**	**Outcome**			**Total**
	**Represent -ativeness**	**Selection of the unexposed cohort**	**Ascertain-ment of exposure**	**Demonstratio n of outcome of interest**	**Comparability of cohorts based on study design**	**Outcome assessment**	**Was follow-up long enough for outcomes to occur**	**Adequacy of follow-up of cohorts**	
Ortiz-Reyes 2022	1	1	1	1	2	1	1	1	9
Jiang 2020	0	0	0	1	1	1	1	1	5
Koga 2018	0	0	0	1	1	1	1	1	5

Table S5. Quality assessment of case-control studies (five studies).

	Selection				Comparability	Exposure		Total
	Case definition adequacy	Representative ness	Selection of controls	Definition of controls	Comparability of cases and controls based on study design	Ascertainment of exposure	Non-response rate	
Liu 2020	1	0	1	1	1	0	0	4
Haac 2018	0	0	1	1	1	0	0	3
Sun 2017	1	0	1	1	1	0	0	4
Patel 2016	1	0	1	1	1	0	0	4
Yuan 2011	1	0	1	1	1	0	0	4

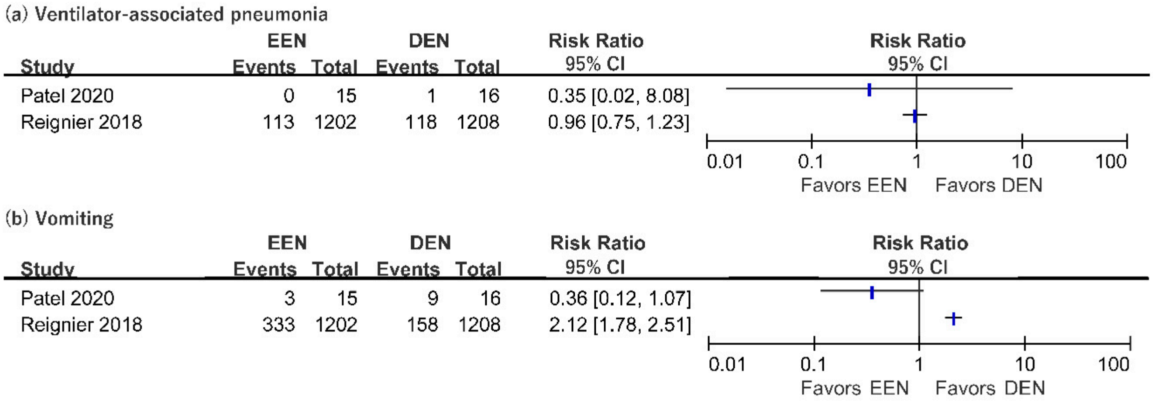

Figure S1. Forest plot of (a) ventilator-associated pneumonia and (b) vomiting events from two RCTs. CI, Confidence Interval; DEN, Delayed Enteral Nutrition; EEN, Early Enteral Nutrition; VAP, ventilator-associated pneumonia.
